# Fluorescence Correlation Spectroscopy Monitors the Fate of Degradable Nanocarriers in the Blood Stream

**DOI:** 10.1021/acs.biomac.1c01407

**Published:** 2022-01-21

**Authors:** Sascha Schmitt, Anne Huppertsberg, Adrian Klefenz, Leonard Kaps, Volker Mailänder, Detlef Schuppan, Hans-Jürgen Butt, Lutz Nuhn, Kaloian Koynov

**Affiliations:** † Max Planck Institute for Polymer Research, Ackermannweg 10, 55128 Mainz, Germany; ‡ Institute for Translational Immunology and Research Center for Immune Therapy, University Medical Center, Johannes Gutenberg University, 55131 Mainz, Germany; § Department of Internal Medicine I, University Medical Center, Johannes Gutenberg-University, 55122 Mainz, Germany; ∥ Department of Dermatology, University Medical Center, Johannes Gutenberg-University, 55122 Mainz, Germany; ⊥ Division of Gastroenterology, Beth Israel Deaconess Medical Center, Harvard Medical School, 02115 Boston, Massachusetts, United States

## Abstract

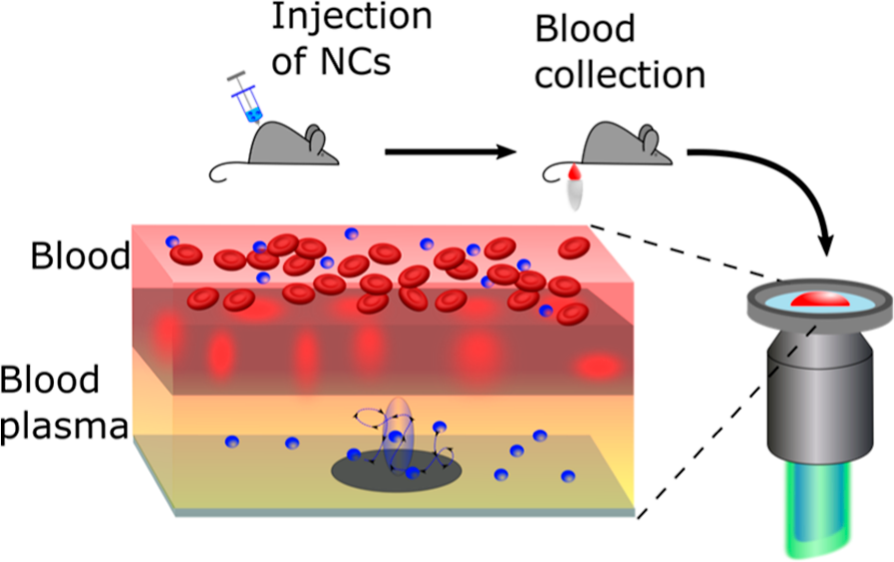

The use of nanoparticles as carriers to deliver pharmacologically active compounds to specific parts of the body via the bloodstream is a promising therapeutic approach for the effective treatment of various diseases. To reach their target sites, nanocarriers (NCs) need to circulate in the bloodstream for prolonged periods without aggregation, degradation, or cargo loss. However, it is very difficult to identify and monitor small-sized NCs and their cargo in the dense and highly complex blood environment. Here, we present a new fluorescence correlation spectroscopy-based method that allows the precise characterization of fluorescently labeled NCs in samples of less than 50 μL of whole blood. The NC size, concentration, and loading efficiency can be measured to evaluate circulation times, stability, or premature drug release. We apply the new method to follow the fate of pH-degradable fluorescent cargo-loaded nanogels in the blood of live mice for periods of up to 72 h.

## 1 Introduction

The use of nanocarriers (NCs) to deliver small drug molecules, proteins, or nucleic acids is a highly promising therapeutic approach. NCs can protect their cargo from the environment during transport through the blood system, deliver it to a target site, and reduce potential side effects from toxic drug molecules.^1−4^ Thus, NC-based drug delivery offers unique potential in fields ranging from treatment of cancer,^5−8^ autoimmunity,^9−11^ and fibrosis^11−15^ to antiviral vaccine development.^16,17^ However, despite the huge research efforts in the last decades, only a moderate number of NCs have entered clinical trials and even fewer are now frontline therapeutics.^4,18^

One of the major challenges in the development of efficient NC-based drug delivery strategies is the need for precise knowledge and control of the NCs’ characteristics such as their size, surface functionalization, drug loading efficiency, and stability during the entire delivery process.^19^ Under aqueous buffer conditions—that is after preparation of the NCs and during their shelf life—these parameters can be monitored relatively easily using a number of experimental techniques including dynamic light scattering, nanoparticle tracking analysis, size exclusion chromatography, zeta potential, and transmission electron microscopy (TEM).^20,21^ However, the situation changes dramatically after the NCs are injected into the blood stream of experimental animals or patients where classical characterization methods typically fail. Sufficient nanoparticle integrity and prolonged NC stability in the blood stream are key prerequisites for NCs if there is to be reliable therapeutic benefit.^22,23^ The high concentration of cells, numerous proteins, and other components in the blood can strongly affect the NCs, for example, by the formation of a protein corona,^24,25^ aggregation, decomposition, or premature release of the drug cargo. It is, therefore, crucial that we are able to track the fate of systemically administered NCs, especially during their circulation in the blood. Given the very small size of the usual NCs (between 10 and 100 nm), the high complexity of the blood components, and variant mechanical forces, this has to date remained a challenging task.

Some techniques, such as computer tomography,^26^ magnetic resonance imaging,^27^ or fluorescence imaging^12,13,28−30^ can be applied in vivo to prove the presence of (labeled) NC in the blood system and provide important information on their body distribution.^12,13,28^ However, these techniques cannot track the NCs’ aggregation, decomposition, or premature release of the drug cargo.

Alternatively, the fate of the NCs in the blood system can be followed in ex vivo blood samples, taken from experimental animals or patients at regular time intervals after NC injection. The NCs in the blood or blood plasma, obtained after a centrifugation step, can then be characterized. The latter approach, which is commonly used, for example, to quantify the concentration of different soluble or microdispersed blood components, is not-well suited for NC characterization for two main reasons. First, the strong shear forces present during the centrifugation step can cause aggregation, decomposition, or loss of drug cargo, particularly in soft carriers such as micelles, nanogels, lipoplexes, or polyplexes. Second, such an approach requires relatively large blood sample amounts (∼mL quantities) that cannot be obtained at regular time intervals from experimental rodents and are unpleasant for vulnerable patients.

Therefore, the only viable option is to characterize the NCs directly in a full blood sample, that is, without prior removal of the blood cells by centrifugation. However, this is difficult because whole blood is a highly crowded, strongly absorbing, and scattering medium. To date, there are only a limited number of reports in the literature on such studies. For example, Carril et al. used ^19^F diffusion-ordered NMR spectroscopy to closely follow the formation of a protein corona on ^19^F-labelled gold nanoparticles in a blood sample.^31^ Braeckmans et al. used single-particle tracking to monitor fluorescent NCs diffusing in the plasma supernatant on top of sedimented blood cells to follow the aggregation of NCs in a small volume sample of whole blood.^32^ Unfortunately, single-particle tracking cannot follow the diffusion of very small particles or released drug cargo molecules. This, however, can be achieved using the fluorescence correlation spectroscopy (FCS) technique, which is sensitive enough to monitor the diffusion of individual fluorescent molecules at nanomolar concentrations.^33^

FCS measures the fluorescent intensity fluctuations caused by fluorescent species diffusing through a very small detection volume, typically the “focus” of a confocal microscope. An autocorrelation analysis of these fluctuations yields information about the diffusion coefficient and the hydrodynamic radius of the fluorescent species, their fluorescent brightness, and concentration.^33^ The species being studied can be single dye molecules, fluorescent (or labeled) drug molecules, proteins, RNA/DNA, or nanoparticles. Nowadays, the FCS technique is an important tool in fields ranging from biology to polymer, colloid, and interface science^34−38^ and has found numerous applications from measuring diffusion in molecularly thin liquids^39^ to quantifying cerebral blood flow.^40^ FCS is particularly well-suited to study drug NC systems in complex media because of its very high sensitivity and selectivity. During the last 2 decades, the technique has often been used to monitor the formation of NCs,^41^ their drug loading,^42,43^ stability,^44−46^ interactions with plasma proteins,^2,42,47−50^ and drug release.^48^ Furthermore, due to the fluorescence-based selectivity of FCS, such studies were performed not only in aqueous buffers but also in blood plasma and other biofluids.^43,48−53^

However, when it comes to whole blood, FCS experiments become exceedingly difficult. This is due to such factors as the strong absorption of light in the visible wavelength range and the large size of the blood cells that can fully occupy the FCS detection volume, thus impeding the diffusion of the fluorescent species through it.

Therefore, only very recently, Negwer et al. presented the first FCS experiments in a sample of whole blood to study the NCs’ size, stability, premature drug release, and interaction with proteins.^54^ To decrease light absorption, the NCs studied and/or their cargo were labeled with dye molecules, with absorption and emission wavelengths in the near-infrared (NIR) range (the so-called biological window). In addition, the experiments were performed during a very slow continuous flow of the blood sample in a microchannel in order to ensure time intervals in which the FCS detection volume is free of blood cells.^54^ Such an approach, however, does not truly represent in vivo conditions. Moreover, it requires NIR labeling and relatively large amounts (1–2 mL) of blood and thus cannot be applied to track ex vivo the behavioral kinetics of the NCs, for example, in a mouse model.

Here, we present a new FCS-based method for characterizing drug NCs and their cargo in whole blood, which overcomes both the need for large quantities of blood and the limitation of using NIR dyes. By preventing the blood cells from entering the FCS detection volume, we can now perform precise FCS experiments with molecular species as small as individual dye molecules in a single droplet of whole blood with volume below 50 μL. This allowed us to follow the fate of a pH-degradable nanogel NC derived from ketal-crosslinked squaric ester amide precursor block copolymers^55^ over 3 days after injection into live mice.

## 2 Materials and Methods

Unless otherwise stated, all chemicals used in this work were purchased from commercial sources, such as Sigma-Aldrich (Taufkirchen, Germany), TCI Chemicals (Tokyo, Japan), or Rapp Polymere (Tübingen, Germany) and used as received. The squaric ester amide methacrylamide (MA-SQ) monomer and the macro chain transfer agent were synthesized following an earlier report.^55^ Fluorescent dyes Alexa 647, Alexa 488, and Oregon Green 488 cadaverine were obtained from Thermo Fisher Scientific (Waltham, MA, USA).

Solvents (HPLC grade) were purchased from Acros Organics (Geel, Belgium) and Fisher Scientific. Millipore water was prepared using a Milli-Q Reference A+ System. For dialysis, Spectra/Por7 dialysis membranes obtained from Spectrum Labs with a molecular weight cutoff of 1000 g mol^–1^ were used.

GR grade Vivid plasma separation membrane was purchased from Pall Corporation (Port Washington, NY 11050). TEM grids with a mesh size of 100 (Standardnetzchen “Pyser”) were purchased from, Plano GmbH, Wetzlar, Germany.

Human blood plasma and human blood were collected and handled according to the guidelines of the ethics committee of the Landesärztekammer Rheinland-Pfalz. Human blood was obtained from a volunteer male donor. The blood was collected in a Li-Heparin-coated tube (Sarstedt, Nümbrecht, Germany) to prevent clotting and was used immediately or stored at 4 °C.

FCS experiments were performed using a commercial confocal microscope (LSM 880, Carl Zeiss, Jena, Germany) equipped with a C-Apochromat 40×/1.2 W (Carl, Zeiss, Jena, Germany) water immersion objective. An argon laser (λ = 488 nm) and a HeNe laser (λ = 633 nm) fiber coupled to the LSM 880 were used for the excitation of the Oregon Green and the Alexa 647 dyes, respectively. The emission light in the spectral range 508–562 nm (Oregon Green) and 655–699 nm (Alexa 647) was detected using a spectral detection unit (Quasar, Carl Zeiss) that comprises a diffraction grating and a multianode photomultiplier operating in the photon counting mode. An Attofluor stainless steel chamber (Thermo Fisher Scientific, Waltham, MA, USA) holding a glass coverslip was used as the sample cell. For the FCS experiments with dyes or NCs in water, phosphate-buffered saline (PBS) buffer, and blood plasma, about 50 μL solution was placed directly on the coverslip. The confocal detection volume was placed ∼15 μm above the glass coverslip. Such short penetration is important in order to diminish the spherical aberration effects caused by the higher (compared to water) refractive index of human plasma, as discussed in details and illustrated in Figure S6 of our previous study.^54^

For the experiments with blood droplets, the following arrangements were used. A round slice of plasma separation membrane was placed in the Attofluor chamber. A TEM grid with a thickness of ∼20 μm was sandwiched between the separation membrane and the supporting coverslip and served as a spacer. The Attofluor chamber was mounted above the microscope objective and a droplet of blood (∼30 μL) was carefully placed on top of the plasma separation membrane. Using confocal imaging in the reflection mode, the TEM grid was localized and the confocal detection volume positioned next to it and ∼15 μm above the glass coverslip. At this position, FCS autocorrelation curves were recorded for 200 s in repetitions of 10 s.

These experimental autocorrelation curves were fitted with the analytical expression of eq 1 (Results and Discussion) using the ZEN software (Carl Zeiss, Jena, Germany). Triplet component with τ_T_ in the range 2–4 μs was used when fitting the curves measured for Alexa 647 (Figure 2A) and the unimers (Figure 5). No triplet component was used when autocorrelation curves of the multiple dye-labeled NCs were fitted. Repetitions, affected by the rare presence of large aggregates, were not considered in the fitting.

**Figure 1:**
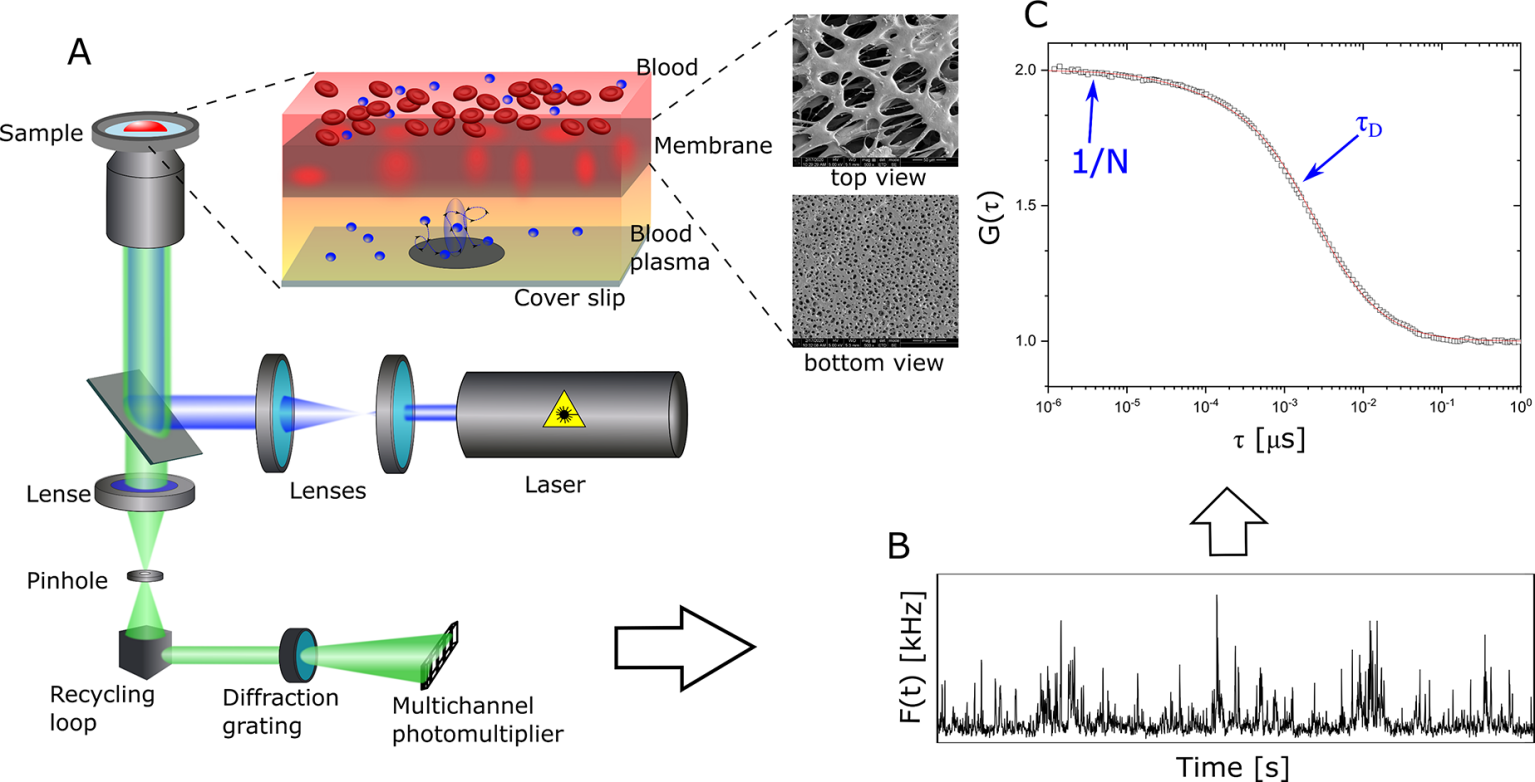
Schematic representation of the FCS experiments and the data processing steps. (A) Optical setup and sample chamber. The inset shows how a membrane prevents the blood cells from reaching the detection volume. The scanning electron microscopy (SEM) pictures illustrate the top view and the bottom view of the membrane. (B) Fluorescence intensity fluctuations caused by the diffusion of the NCs through the detection volume. (C) Autocorrelation curve (black symbols) derived from (B) and the corresponding fit (red line) with eq 1. Blue symbols show parameters obtained from the fit.

**Figure 2:**
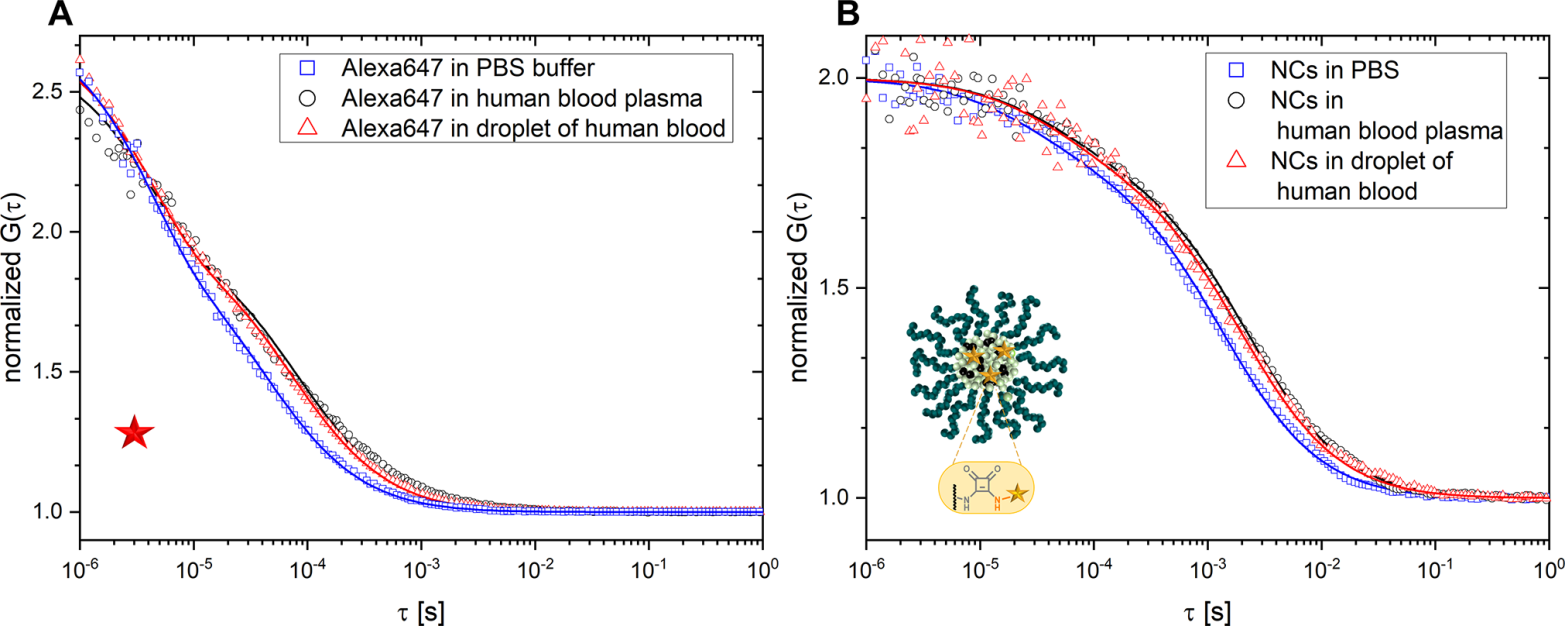
Initial tests of the FCS in a blood droplet method. (A) Normalized autocorrelation curves for Alexa 647 measured in a droplet of heparin-treated human blood (red triangles), human blood plasma (black circles), and PBS (blue squares). (B) Normalized autocorrelation curves for squarogel NCs measured in a droplet of heparin-treated human blood (red triangles), human blood plasma (black circles), and PBS (blue squares). The solid lines represent the corresponding fits obtained from eq 1.

**Figure 3:**
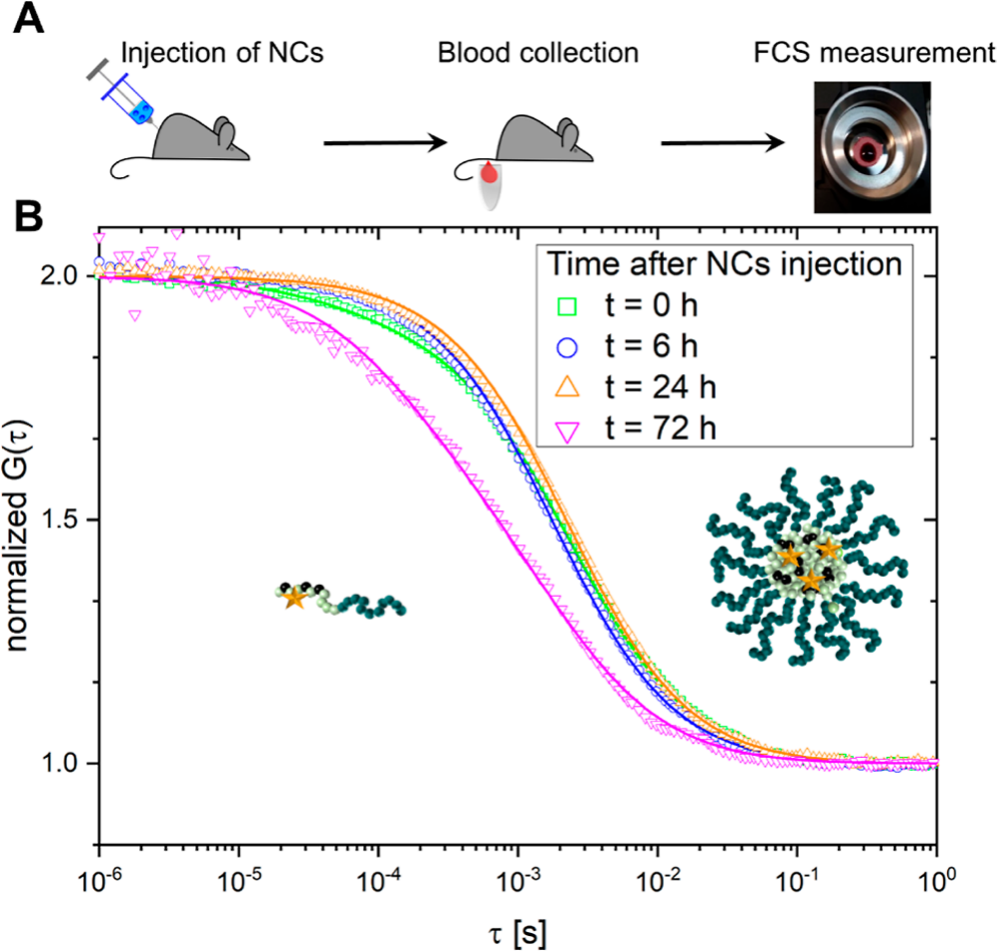
Monitoring the fate of squarogel NCs in the blood stream after intravenous injection into a mouse. (A) Schematic overview of the experimental procedure. (B) Normalized autocorrelation curves recorded in blood samples taken from a mouse 0 (green), 6 (blue), 24 (orange), and 72 h (magenta) after injection of NCs.

**Figure 4:**
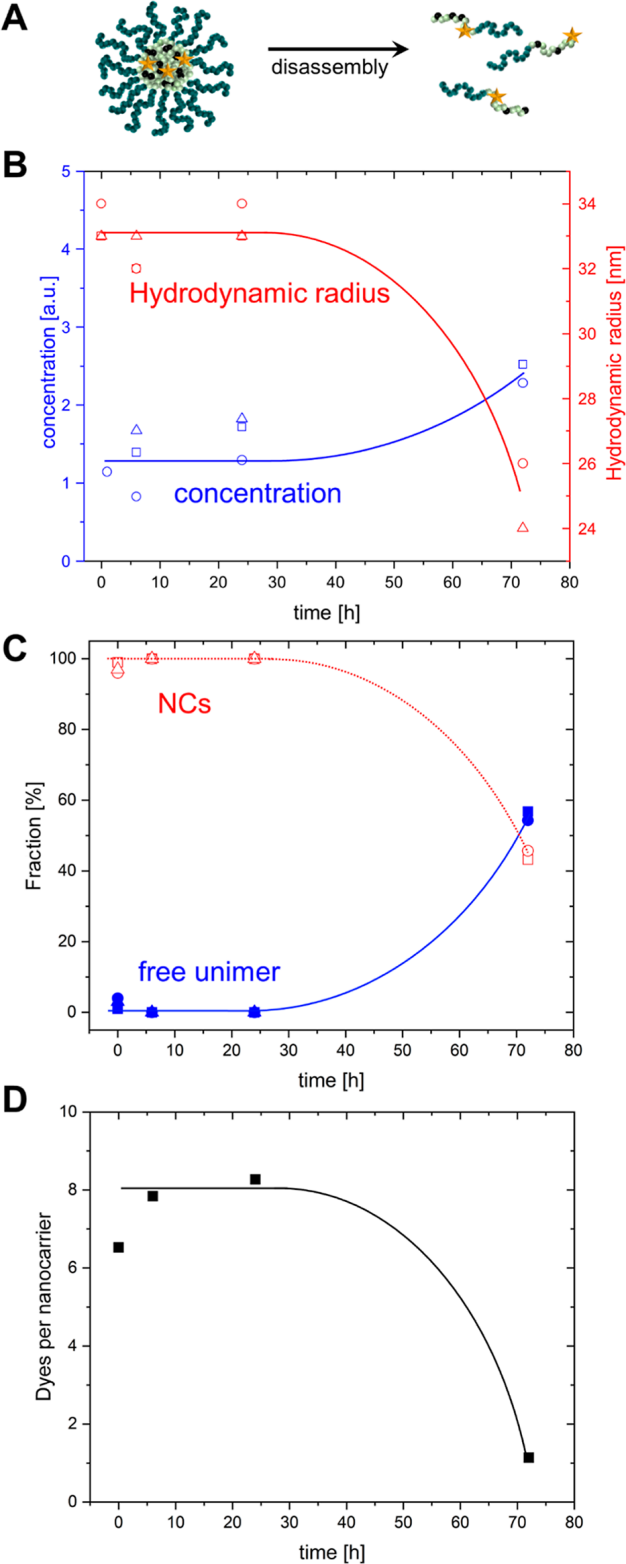
Quantifying the fate of squarogel NCs in the blood stream after intravenous injection into a mouse. (A) Schematic of the squarogel decomposition. (B) Average hydrodynamic radius of the NCs (red symbols) and overall concentration of the diffusing fluorescent species (blue symbols) vs time after injection. (C) Relative fractions of NCs (open symbols) and free unimers (closed symbols, representing single polymer chains of degraded nanogels) vs time after injection. The data presented in (B,C) were obtained from three independent mouse experiments. (D) Average number of dye molecules per NC vs time after injection. The solid lines in (B–D) are a guide to the eye.

**Figure 5:**
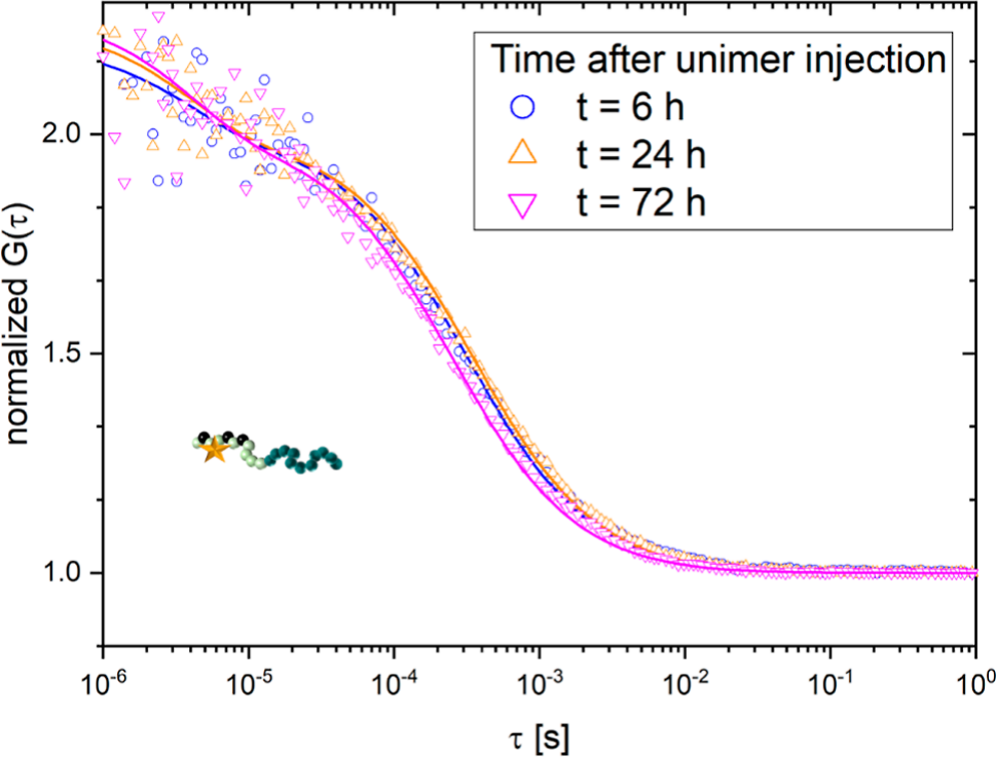
Monitoring the fate of non-crosslinked polymers (unimers) in the blood stream of a live mouse. Normalized autocorrelation curves recorded in blood samples taken from a mouse 6 h (blue), 24 h (orange), and 72 h (magenta) after injection of unimers.

In a FCS experiment, the size of the confocal detection volume depends on a number of parameters including the used microscope objective and the wavelengths of the excitation and emission. Therefore, in order to obtain quantitative information for the diffusion coefficients and the hydrodynamic radii of the studied species, the size of the detection volume has to be determined beforehand. To this end, we performed calibration FCS experiments with dyes with known diffusion coefficients, namely, Alexa Fluor488 and Alexa Fluor647 dissolved in Milli-Q-water.

The conducted animal studies followed the approval of the local ethics committee on animal care (number 23 177-07/G 17-1-030, Government of Rhineland Palatinate, Germany). 8–10 week-old female BALB/c mice (body weight ∼20–25 g) were bought from Charles River (Sulzfeld, Germany) and kept according the guidelines of the institute and the local government (12 h light–dark cycles at 25 °C and 40–60% humidity with humane care, access to regular chow and water ad libitum). Mice were injected with 100 μL of a 2 mg mL^–1^ solution intravenously with nanoparticles or monomers, respectively. Blood samples (50 μL) were taken via puncture of the submandibular vein after 0, 6, 24, and 72 h.

## 3 Results and Discussion

Our experimental setup for studying NCs in droplets of whole blood is schematically represented in Figure 1. It uses a classical confocal FCS arrangement based on a commercial confocal microscope, as described in the Materials and Methods section. Briefly, a high numerical aperture microscope objective focuses an excitation laser beam into the studied blood sample (placed on a microscope coverslip) containing the fluorescent species. The emitted fluorescent light is collected by the same objective, passed through a confocal pinhole, and directed to a sensitive photodetector. This arrangement results in the formation of a small (<1 μm^3^) confocal detection volume. Only fluorescent light from fluorophores that are in the detection volume can reach the photodetector. The diffusion of the fluorescent species through the detection volume leads to temporal fluctuations in the detected fluorescence intensity *F*(*t*) that are recorded and processed in the form of an autocorrelation function . Here, δ*F*(*t*) = *F*(*t*) – ⟨*F*(*t*)⟩ and ⟨ ⟩ denotes the time average. For an ensemble of *m* types of fluorescent species freely diffusing through a detection volume with a three-dimensional Gaussian shape, the autocorrelation function has the following analytical form^33^
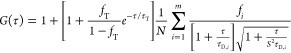
1*f*_T_ and τ_T_ are the fraction and the decay time of the triplet state, *N* represents the average number of fluorescent species diffusing through the observation volume and, thus, it is directly proportional to their concentration. τ_D,*i*_ is the diffusion time of the *i*-th type of species, *f*_*i*_ is their fraction, and *S* is the so-called structure factor *S* = *z*_0_/*r*_0_, where *z*_0_ and *r*_0_ represent the axial and radial dimensions of the confocal volume, respectively. The diffusion time τ_D,*i*_ relates to the diffusion coefficient *D*_*i*_ through *D*_*i*_ = *r*_0_^2^/4τ_D,*i*_. By fitting the experimentally measured autocorrelation curves with eq 1 one can obtain the diffusion coefficients, and consequently, the hydrodynamic radii *R*_H_ of the fluorescent species through the Stokes–Einstein relation as *R*_H_ = *k*_B_*T*/6πη*D*, where *T* is the absolute temperature, *k*_B_ the Boltzmann constant, and η the viscosity of the solvent. Last, the average fluorescence brightness (FB) of the studied species can be evaluated by dividing the average fluorescent intensity ⟨*F*(*t*)⟩ to the average number of this species in the observation volume *N*, that is, FB = ⟨*F*(*t*)⟩/*N*. This parameter is important because by comparing the fluorescent brightness of a model fluorescent drug molecule to the fluorescent brightness of the NC loaded with this drug, one can estimate the drug loading efficiency and monitor triggered or premature release.

However, when the confocal detection volume is positioned directly into a droplet of full blood, it will be partially or fully occupied by sedimented blood cells, which prevents recording of FCS autocorrelation curves. As shown in our earlier work,^54^ even the use of NIR excitation and the active search of “free” spots between the sedimented cells cannot solve this problem. Therefore, here, we used a porous structure that allows the liquid part of the blood as well as the NCs to pass through but prevents the larger blood cells from entering the detection volume during FCS measurement (Figure 1A). After characterizing several commercially available membrane structures, we found that the Vivid plasma separation membranes were particularly suitable and effective. They consist of an asymmetric porous polysulfone structure and have high plasma yield, low analyte binding, and a low hemolysis tendency.^56^ In our experiments, a blood droplet was placed on a ∼10 mm diameter disk-shaped piece of membrane supported by a TEM grid serving as a spacer. The confocal detection volume was positioned in the space between the microscope cover slip and the membrane (Figure 1A).

Alexa 647 as standard fluorescent dye commonly employed in FCS experiments was used in initial trials of these FCS settings, analyzing small blood droplets. To this end, Alexa 647 was dissolved in heparin-treated human blood to a final concentration of 10 nM L^–1^. After 30 min of incubation, a 30 μL blood droplet was placed on top of the plasma separation membrane and studied immediately by FCS. A typical autocorrelation curve is shown in Figure 2A by red symbols. Autocorrelation curves for Alexa 647 dissolved in blood plasma (black symbols) and PBS buffer (blue symbols) measured in the conventional way (without plasma separation membrane) are also shown for comparison. It is evident that the autocorrelation curves measured both in a droplet of whole blood and in blood plasma are nearly identical. Both curves could be fitted with a single component fit (eq 1, *m* = 1) that yielded almost equal diffusion times of τ_blood_ = 71 ± 4 μs and τ_plasma_ = 69 ± 4 μs. In contrast, the fit of the autocorrelation curve measured in PBS yielded a faster diffusion time, τ_PBS_ = 45 ± 2 μs. The difference in the diffusion times arises from the roughly 1.5-fold higher viscosity of blood plasma compared to PBS.^54^ After calculating the diffusion coefficient of the Alexa 647 dye in all three studied media and accounting for the different viscosities in the Stokes–Einstein relation, similar values of *R*_H_ ≈ 0.75 nm could be obtained as hydrodynamic radii of Alexa 647 in PBS, blood plasma, as well as whole blood. These results indicate that in accordance with earlier studies,^57^ Alexa 647 dye molecules are inert and do not interact with plasma proteins. More importantly, the results confirmed that the newly developed FCS in a blood droplet method can be successfully used to monitor and characterize species even as small as individual dye molecules.

After testing the new FCS in a blood droplet method with small dye molecules, we opted for a biodegradable drug NC system with potential applications for immunodrug delivery and reasonable long blood circulation times. Among the various types of block copolymer-based nanogels already developed by Nuhn et al.,^12,58−61^ blood-stable nanogels derived from amine-reactive methacrylamides with pendant squaric ester amides were introduced recently.^55^ They are fabricated from RAFT-polymerized PEG block copolymers that are self-assembled into precursor micelles and sequentially transformed by amine-bearing acid-sensitive ketal crosslinkers, drugs, dyes, and short oligo(ethylene glycol)s into fully hydrophilic nanogels (Supporting Information, Figures S1–S6). Due to their high degree of PEGylation, these squaric ester-based nanogels (so-called “squarogels”) provide profound stability in human plasma while being equipped with a stimuli-responsive degradation property upon exposure to endolysosomal pH conditions (Supporting Information, Figure S7). Intravenously injected squarogels circulate in the blood stream with an even distribution all over the body and progressive accumulation mainly in the liver, spleen, or kidney.^55^

We decided to use these carriers in our current study because of their pH-responsive degradation profile; thus, they can undergo a transition from being an intact nanogel into being single soluble polymers upon hydrolysis of the acid-sensitive ketal crosslinker (Figure 4A). Intact nanogels as well as non-crosslinked polymers representing the degraded nanogel version can be covalently loaded with Oregon Green dye as a model for drug cargo that also serves as fluorescent label for FCS monitoring. Further details on synthesis and specification of these NCs can be found in the Supporting Information.

Typical autocorrelation curves of the squarogel NCs, recorded in PBS buffer, human blood plasma, and in a droplet of heparin-treated human blood are shown in Figure 2B. For all three sets of experiments, the NCs were incubated for 30 min in the respective media prior to performing the FCS measurements. The experimental curves were fitted with eq 1 to obtain the respective diffusion times. In all cases, a two-component fit was needed (*m* = 2 in eq 1) to account for a certain fraction (∼20%) of free, non-bound Oregon Green dye molecules that were still present in the squarogel sample being studied, even after extensive purification steps (Supporting Information). The NCs showed a diffusion time of τ_PBS_ = 1400 ± 60 μs in PBS buffer, which corresponds to an average hydrodynamic radius of *R*_H,NCs_ = 32 ± 3 nm. The diffusion times obtained in blood plasma τ_plasma_ = 2200 ± 160 μs and in a droplet of blood τ_blood_ = 2100 ± 250 μs translate to hydrodynamic radii of *R*_H,NCs,plasma_ = 33 ± 3 nm and *R*_H,NCs,blood_ = 31 ± 4 nm after accounting for the plasma viscosity. Moreover, the average number of particles in the FCS detection volume was similar for blood plasma, whole blood, and PBS reflecting comparability of the respective NC concentrations. These results confirm that passage through the plasma separation membrane did not affect the NCs, such as reducing their concentration or inducing degradation or aggregation. Furthermore, the fact that the hydrodynamic radius did not change significantly in whole blood and blood plasma versus PBS indicates minor interactions between plasma proteins and the squarogel NCs. These findings correspond with previously observed properties of the squarogels, such as poor uptake by phagocytes and other cells, and long residence in the blood stream, properties that can be correlated to their high degree of PEGylation.^55^

Next, we decided to take full advantage of the newly developed FCS in a blood droplet method by using it to monitor the fate of the squarogel NCs after their intravenous injection into live mice. 8–10 week-old female BALB/c mice (body weight ∼20–25 g) received 100 μL of 2 mg mL^–1^ squarogel NCs or non-crosslinked polymers (unimers) representing degraded NCs. Next, blood samples (50 μL) were taken via puncture of the submandibular vein after 0 h (2–5 min), 6, 24, and 72 h. The blood samples collected were anticoagulated with heparin and stored in regular Eppendorf tubes. FCS measurements were carried out less than 1 h after collection of the blood samples. A schematic overview of the experimental procedure is shown in Figure 3A.

Typical normalized autocorrelation curves recorded in samples taken from one mouse (Figure 3B) illustrate the kinetics of the NCs’ stability during their circulation in the blood stream. The curve recorded in the sample taken shortly after injection (“time 0 h”) shows two decays and needs to be fitted with a two-component fit (*m* = 2 in eq 1). The fast decay reflects the free, non-bound Oregon Green dye that was initially present as small impurity in the injected NC suspension, while the slower decay originates from the covalently dye-labeled NCs. Interestingly, the curves recorded in the samples taken 6 and 24 h after injection showed only one slow decay and could be well-fitted by single-component fits.

It is known that species smaller than 5 nm are rapidly cleared from the blood vessel system, mainly via the kidneys.^1^ Our FCS experiments nicely confirmed that unbound Oregon Green molecules are cleared from the body quite fast, in less than 6 h. The fits of all three curves (samples taken 0, 6, and 24 h after injection) showed that the diffusion time and thus the respective hydrodynamic radius of the NCs remained unchanged at *R*_H,NCs,mouse_ = 33 ± 3 nm within the first 24 h. We concluded from these results that neither aggregation nor degradation of the NCs occurred in the first 24 h of circulation in the blood system of a live mouse. Furthermore, the concentration of NCs in the blood (Figure 4B, blue symbols) did not change considerably during the first 24 h either. However, after 72 h of circulation, we observed signs of NC degradation as illustrated by the overall shift of the corresponding autocorrelation curve (Figure 3B) to shorter lag times, indicating a decrease in the size of the observed fluorescence species. Furthermore, this curve needed to be fitted by a two-component fit, yielding two diffusion times. The longer diffusion time originated from partially degraded squarogel NCs still circulating in the blood stream and provided a hydrodynamic radius of *R*_H,NCs,mouse,72h_ = 24 ± 2 nm. The shorter diffusion time originated from a smaller species with hydrodynamic radii of 5 ± 1 nm, corresponding to individual block copolymer molecules that appeared upon hydrolysis of the ketal crosslinks inside the squarogel NCs. This was further supported by an increase in the average concentration of the diffusing fluorescent species (Figure 4B, blue symbols).

The unfolding of the squarogel NCs and the release of multiple-labeled unimers led to an overall increase in the concentration of the fluorescent species in the blood. Moreover, these FCS experiments allowed us to determine the loading efficiency of the Oregon Green molecules (as model for drug cargo) in the NCs. The data shown in Figure 4D indicate that the NCs were loaded with average of 7–8 cargo molecules and retained their degree of cargo loading in the first 24 h of circulation in the blood stream. Only after 72 h, the loading efficiency decreased significantly as a result of the NCs’ decomposition. Note that the values reported in Figure 4D should be considered with care because the slightly lower signal-to-noise ratio in the blood measurements (due to autofluorescence background of around 10% compared to NC signal) and the presence of fluorescent species with low brightness (free dye at *t* = 0 h and unimers at *t* = 72 h) can result in overestimation of the value of *N* for the NCs and, thus, an underestimation of the loading efficiency.

Taken together, 72 h after their injection the NCs were still circulating in the mouse blood stream, while their partial degradation was evident by a 30% decrease in their size, decrease in loading efficiency, and the release of fluorescently labeled unimers. Interestingly, it appears that these rather small unimers with *R*_H,unimer_ = 5 nm were not yet fully cleared from the blood. To confirm these findings, we injected these unimers into the blood stream of a mouse in an independent experiment and studied their fate again using the newly established FCS approach. The autocorrelation curves obtained from blood samples taken after 6, 24, and 72 h (Figure 5) were almost identical and yielded a diffusion time τ_unimer,mouse_ ≈ 300 ± 30 μs that corresponded to a hydrodynamic radius of *R*_H,unimer,mouse_ = 5.1 ± 1 nm. This value is in accordance with the one measured for the unimers in PBS buffer *R*_H,unimer,PBS_ = 5.8 ± 1 nm and indicates that the unimers were still circulating in the mouse’s blood stream even 72 h after injection. This can also be explained by the high degree of PEGylation of these hydrophilic block copolymers which can afford longer circulation times to achieve clearance from the body than other polymers.

Last, we were interested in the mechanistic background of the partial degradation which we observed in the squarogel NCs during their long circulation. Possible reasons for degradation can be related to the shear forces that the NCs experience during their flow through the murine blood vessels and to the elevated temperature compared to normal storage conditions. Thus, we excluded the former and tested the elevated temperature by simulating the in vivo conditions in a cuvette in vitro. We incubated the ketal-crosslinked squarogel NCs in either full human blood orblood plasma at 4 and 37 °C and recorded their integrity by FCS experiments over 72 h. At 4 °C, the NC provided high stability and did not show any signs of degradation in both media (Supporting Information, Figure 8A,C). This was also the case for the squarogels incubated at 37 °C up to 24 h (Supporting Information, Figure 8B,D). Only at 72 h, we were able to observe a similar gradual NC disintegration and release of individual block copolymers in analogy to the previously obtained measurements from the samples injected into the blood stream of live mice (Figure 4). Remarkably, at this late time, nanogel unfolding is observed not only in whole blood or plasma but also in PBS (Supporting Information, Figure 8E). These results demonstrate that the ketal crosslinks provide transient stability under physiological pH and temperature, but undergo gradual nanogel disassembly after 24 h. Apparently, this is related to the hydrolytic sensitivity of the ketal group^60,62^ both in PBS at 37 °C, and during blood circulation in the live mice (Figure 4). Our newly developed FCS in a blood droplet method allowed us to correlate these in vitro findings with in vivo changes that occur over intervals of time.

## 4 Conclusions

In conclusion, we have developed an FCS-based method that allows for the monitoring of fluorescent NCs and their cargo down to the size of individual fluorescent molecules in droplets of undiluted whole blood samples. The size, the concentration, and the loading efficiency of NCs dispersed in the liquid part of the blood at the time of the experiment can be measured. NCs that stick to blood cells or are endocytosed by, for example, cells of the reticuloendothelial system (macrophages/monocytes) can of course not be characterized directly, but through the decrease of their concentration in the liquid part of the blood, their disappearance during circulation can be concluded indirectly. The experiments can be performed on commercial FCS equipment and with common fluorophores that may have any excitation wavelength in the visible or NIR range. By applying this method, we were able to monitor, with unprecedented sensitivity, the fate of degradable NCs in the bloodstream of mice by repetitive sampling of very small volumes of blood (50 μL) from a live mouse at defined time intervals.

This FCS method allowed us to quantify the integrity over time of a ketal-crosslinked nanogel system derived from squaric ester amide block copolymers (squarogels) in the blood stream after intravenous injection. We observed that a minor fraction of non-conjugated dye impurities was effectively cleared from the blood vessel system in less than 6 h, while the NCs remained stable and intact, circulating in the blood stream for up to 24 h. Only after 24–72 h, a partial disassembly into single soluble block copolymers with reduced sized was observed, which continued to circulate in the blood stream. These observations obtained from samples of live mice were supported by in vitro incubation studies in whole blood, plasma, or PBS and confirm the hydrolytic sensitivity of the ketal group at physiological pH and temperature over time.

We anticipate that the FCS in blood droplets method presented here, which enables accurate and quantitative monitoring of the behavior of NCs in vivo, will provide new opportunities to monitor the NCs’ integrity during blood circulation and, thus, contribute toward the development of improved NC-based therapeutics with transient stability and predictable biodegradation.

## Supporting Information Available

Details on the synthesis of the nanogel precursors and the nanogels; characterization of the nanogels; and FCS autocorrelation curves for NCs incubated in PBS, blood plasma, and whole blood (PDF)
