# Supplementary material for: Fluorescence Correlation Spectroscopy Monitors the Fate of Degradable Nanocarriers in the Blood Stream
Source: Biomacromolecules. 2022 Jan 21;23(3):1065–74. doi: 10.1021/acs.biomac.1c01407 (PMC8924869; doi:10.1021/acs.biomac.1c01407)
Supplement: Supplementary file 1 — bm1c01407_si_001.pdf [file bm1c01407_si_001.pdf]

Microscale Fluorescence Correlation Spectroscopy to Monitor the Fate of Degradable Nanocarriers in the Blood Stream

Supporting Information

Sascha Schmitt[1], Anne Huppertsberg[1], Adrian Klefenz[2], Leonard Kaps[2,3], Volker Mailänder,[1,4] Detlef Schuppan,[2,5] Hans-Jürgen Butt[1], Lutz Nuhn[1,*], Kaloian Koynov[1,*]

[1]Max Planck Institute for Polymer Research, Ackermannweg 10, 55128 Mainz, Germany

[2]Institute for Translational Immunology and Research Center for Immune Therapy, University Medical Center, Johannes Gutenberg University, Mainz, Germany

[3]Department of Internal Medicine I, University Medical Centre, Johannes Gutenberg-University, Mainz, Germany

[4]Department of Dermatology, University Medical Centre, Johannes Gutenberg-University, Mainz, Germany

[5]Division of Gastroenterology, Beth Israel Deaconess Medical Center, Harvard Medical School, Boston, Massachusetts, United States

Table of content:

1. Additional Characterization Methods 3

2. Nanogel Preparation 4

2.1 Block Copolymer Syntheses 4

2.2 Nanogel Synthesis 7

3. FCS incubation experiments 10

4. References 13

1. Additional Characterization Methods

DLS measurements of the nanogels were performed at 25 °C using a Malvern ZetaSizer Nano S purchased from Malvern Instruments Ltd. (Malvern, Great Britain) with a He/Ne Laser (λ = 633 nm) at a fixed scattering angle of 173°.

^{1}H NMR spectra of the precursor polymes for the nanogel synthesis were recorded at room temperature on a Bruker Avance III 300 MHz spectrometer. The chemical shifts (δ) are given in parts per million (ppm) relative to TMS. NMR spectra were processed with the software MestReNova 11.0.4 by Mestrelab Research. Samples were prepared in deuterated solvents and their corresponding signals referenced to residual non-deuterated solvent signals.

Size-exclusion chromatography of the precursor polymes for the nanogel synthesis was performed using the following set-up: a PU2080+ pump, an auto sampler AS1555, an UV detector UV2075+ and a RI-detector RI2080+ from JASCO. Hexafluoroisopropanol (HFIP) containing 3.0 g L^{-1} of potassium trifluoroacetate was used as eluent at a flowrate of 0.8 mL min^{-1} and a column temperature of 40 °C. The column material was composed of modified silica obtained from MZ-Analysentechnik: PFG columns, particle size: 7 µm, porosity: 100 Å + 1000 Å. A calibration with poly(methyl methacrylate) (PMMA) standards was purchased from PSS (Mainz, Germany) and used for determining relative molecular weight. Toluene was used as internal standard. Samples were prepared at 1 mg mL^{-1} and filtered through PVDF syringe filters (0.2 µm pore size, Acrodisc) prior to injection. The data was processed with the software PSS WINGPC UniChrom.

UV/Vis spectra of the sequential bisamide formation during squarogel fabrication was recorded on a V-630 spectrophotometer equipped with a Peltier thermostatted ETC-717 single cell holder purchased from JASCO (Pfungstadt, Germany). Measurement conditions of 20 °C were guaranteed by a V50 water thermostat from Krüss Optronic (Hamburg, Germany).

2. Nanogel Preparation

2.1 Block Copolymer Syntheses

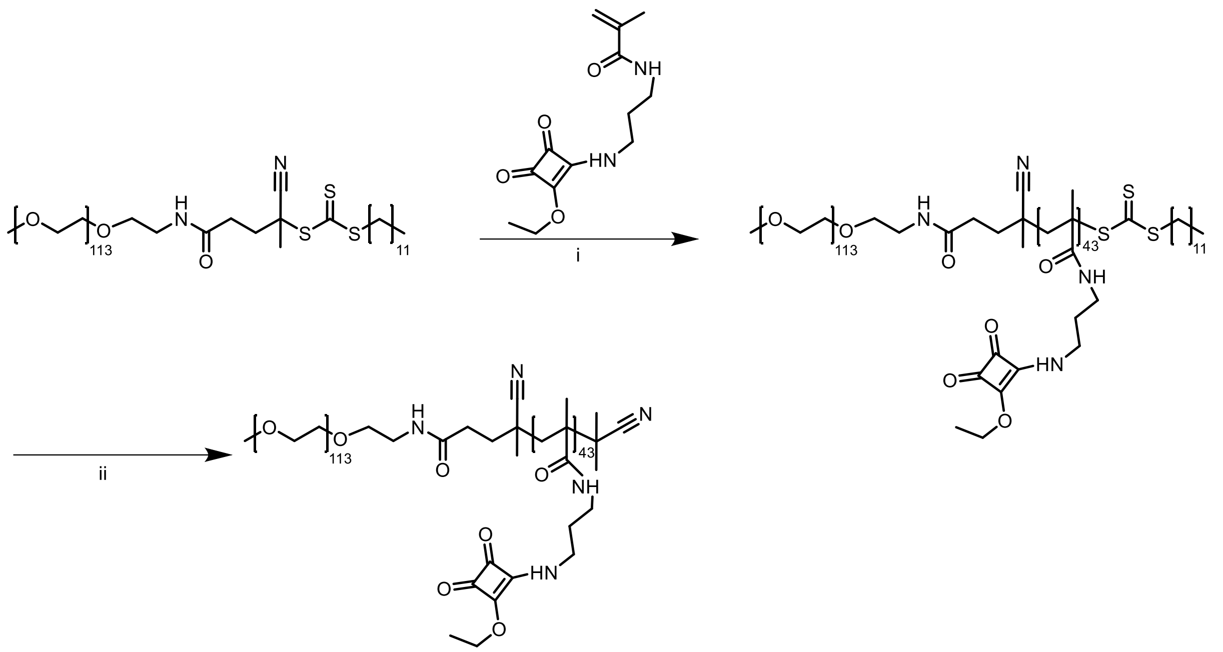

Figure S1. Synthesis of block copolymer mPEG$_{113}$-b-(p(MA-SQ)$_{43}$ with macro-PEG-DDC-TTC-CTA, i) AIBN, DMF, 70 °C, 42 h, yield: quantitative, ii) excess AIBN, DMF, 70 °C, 12 h, yield: quantitative.

As described earlier by Huppertsberg et al. [1], the amphiphilic block copolymer poly(ethylene glycol)-b-poly(N-(3-(2-ethoxy-3,4-dioxocyclobut-1-en-1-yl)amino)propyl)-methacrylamide (mPEG$_{113}$-b-p(MA-SQ)$_n$) is used as precursor for the fabrication of pH-responsive squaric ester-based nanogels. The block copolymer can be obtained by reversible addition-fragmentation chain-transfer (RAFT) polymerization of the monomer N-(3-(2-ethoxy-3,4-dioxocyclobut-1-en-1-yl) amino)propyl)methacrylamide (MA-SQ) using mPEG$_{113}$-dodecyl-trithiocarbonate as chain transfer agent (macro-PEG-DDC-TTC-CTA). The synthesis of the herein used block copolymer was proceeded in analogy and is described in detail in the following (the syntheses of the monomer and macro-CTA can be found here [1].

Block Copolymerization (i)

AIBN (1.3 mg, 8.0 µmol, 0.2 eq.), macro-CTA (200.0 mg, 40.0 µmol, 1.0 eq.), and MA-SQ (533.0 mg, 2.0 mmol, 50 eq.) were loaded into a Schlenk tube equipped with a stirring bar and dissolved in anhydrous DMF (2 mL). The reaction mixture was degassed by three freeze-pump-thaw cycles, before it was immersed into an oil bath at 70 °C for 42 h, while being in its evacuated state. ^{1}H-NMR analysis of a reaction mixture aliquot reported a monomer conversion of 86 % after the reaction time. Isolation

of the block copolymer was afforded by three-fold precipitation in cold diethyl ether (-20° C) followed by centrifugation. After subsequent drying under high vacuum for 12 h mPEG$_{113}$-b-p(MA-SQ)$_{43}$-CTA was obtained as yellow solid (734.8 mg, 44.5 µmol, quantitative).

SEC (HFIP, PMMA-Std.): M_n = 37,600 g mol^{-1} M_w = 41,330 g mol^{-1}, PDI = 1.10.

End group removal (ii)

For the elimination of the macro-CTA derived trithiocarbonate end group, the block copolymer was reacted with an excess of AIBN. Therefore, the block copolymer mPEG$_{113}$-b-p(MA-SQ)$_{43}$-CTA (720.0 mg, 43.6 µmol, 1. eq.) and AIBN (358.0 mg, 2.20 mmol, 50 eq.) were loaded into a Schlenk tube equipped with a stirring bar and dissolved in anhydrous DMF (3 mL) under an inert atmosphere. The reaction mixture was placed into an oil bath at 70 °C for 16 h. Subsequently the block copolymer was isolated by three-fold precipitation into cold diethyl ether (-20° C) and centrifugation. Drying under high vacuum for 14 h afforded mPEG$_{113}$-b-p(MA-SQ)$_{43}$ (570 mg, 34.5 µmol, 79 %) as yellow solid.

^{1}H-NMR (300 MHz, DMSO-d_6): δ (ppm) = 8.74 (s, 0.5H, **a**)$^{a)}$, 8.58 (s, 0.5H, **a**)$^{a)}$, 7.31 (s, 1H, **b**), 4.76-4.54 (m, 2H, **c**),3.83-3.48 (m,10H, **d**), 3.48-3.35 (m, 1H, **e**)$^{a)}$, 3.30-3.17 (m, 1H, **e**)$^{a)}$, 3.17-2.74 (m, 2H, **f**), 1.92-0.48 (m, 10H, **g**). $^{a)}$ due to rotamers a splitting of the signals is observed.

SEC (HFIP, PMMA-Std.): M_n = 37,050 g mol^{-1} M_w =40,310 g mol^{-1}, PDI = 1.09.

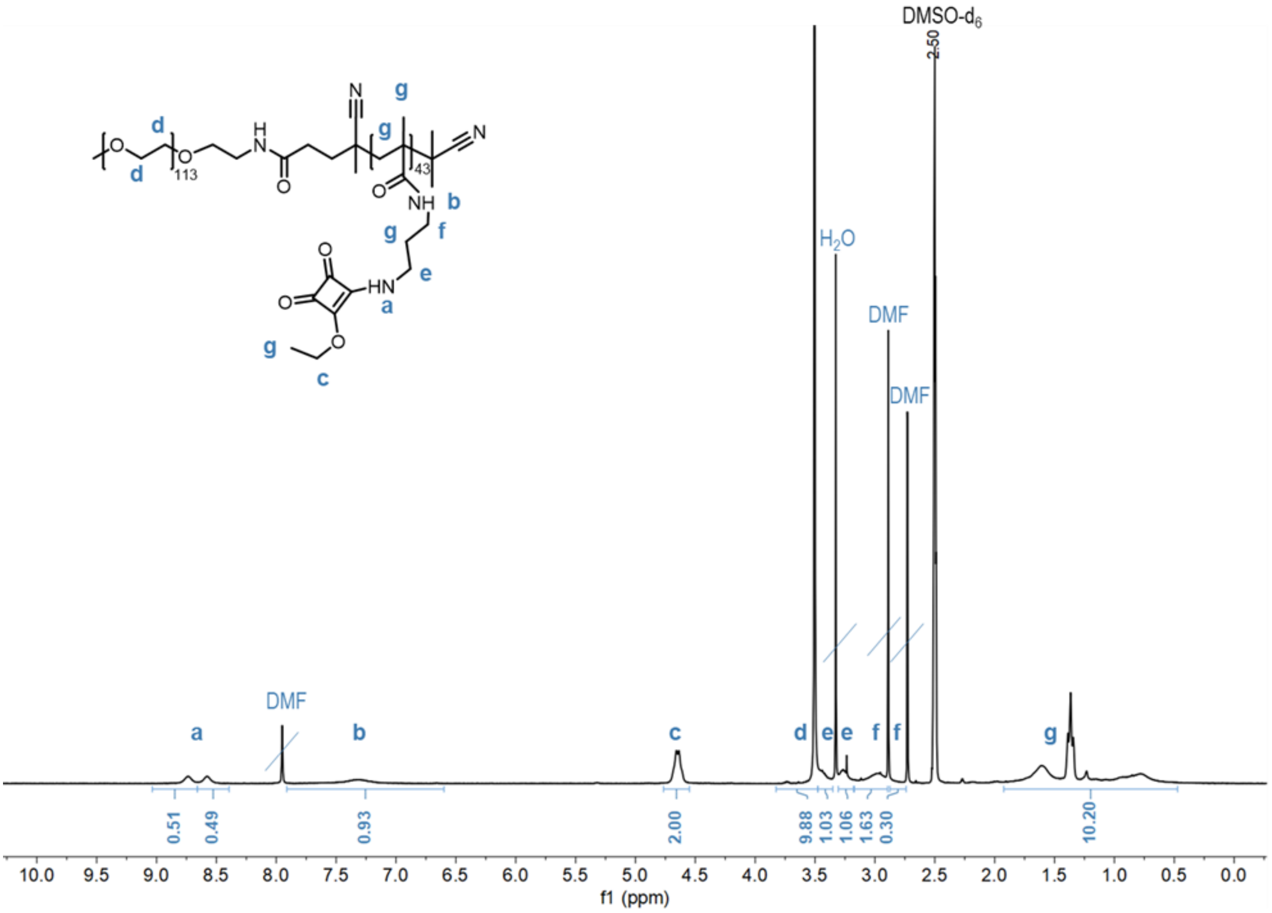

Figure S2. ^{1}H-NMR (300 MHz) spectrum of $mPEG_{113}$-b-p(MA-SQ)$_{43}$ in DMSO-d_6.

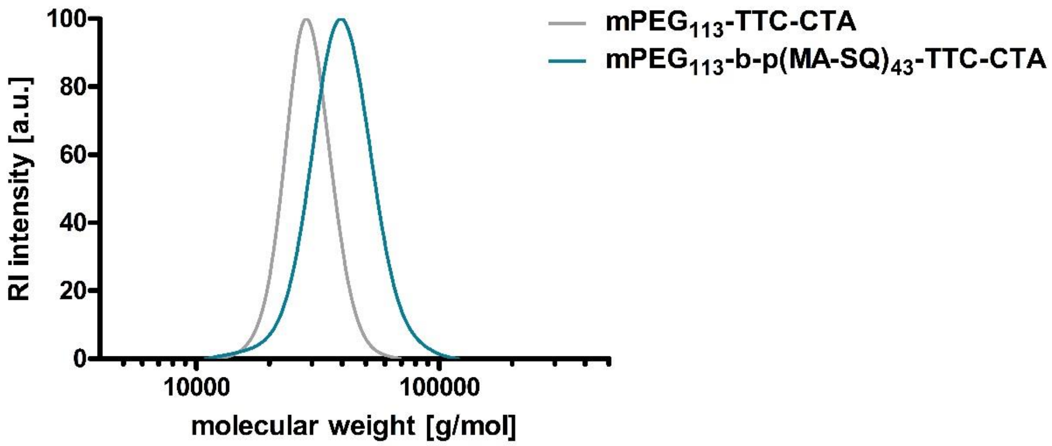

Figure S3. HFIP SEC elugram of the macro chain transfer agent ($mPEG_{113}$-TTC-CTA) (grey) and the corresponding block copolymer $mPEG_{113}$-b-p(MA-SQ)$_{43}$-TTC-CTA before end group removal (blue).

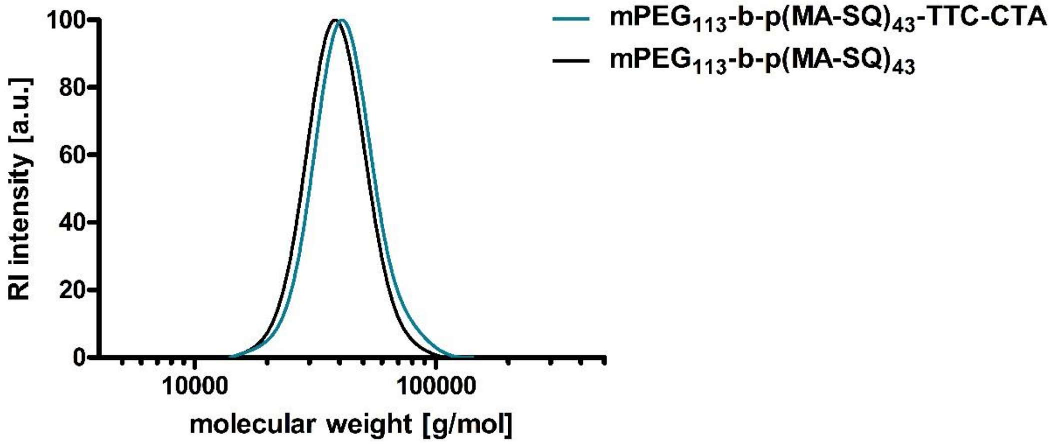

Figure S4. HFIP SEC elugram of the block copolymer $mPEG_{113}$-b-p(MA-SQ)$_{43}$ before (blue) and after end group removal (black).

2.2 Nanogel Synthesis

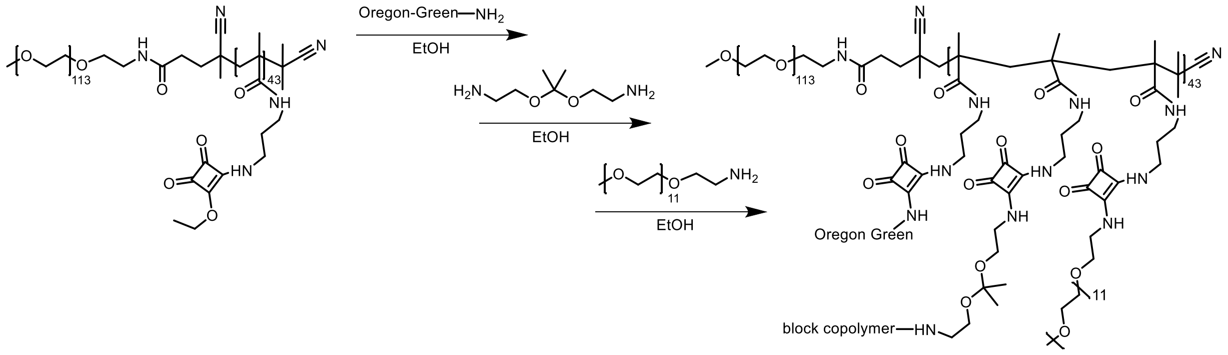

Figure S5. Scheme of sequential preparation of Oregon Green-labeled squarogels.

The preparation of squaric ester-based nanogels (referred to as squarogels) using $mPEG_{113}$-b-p(MA-SQ)$_{43}$ derived block copolymers has been described recently.[1] The herein investigated Oregon-Green-labeled, pH-responsive squarogels have been fabricated in analogy:

Initially, the block copolymer $mPEG_{113}$-p(SQ-MA)$_{43}$ (30.0 mg, 1.89 µmol polymer, 75.56 µmol reactive squaric ester amide units) was dispersed in ethanol at a final concentration of 10 mg/mL. After 1 h of ultrasonicating, the formation of self-assembled polymeric micelles was confirmed by DLS measurements. Next, the micellar solution was transferred into a Schlenk tube equipped with a stirring bar and 150 µL of Oregon Green cadaverine stock solution (2.5 mg mL^{-1} in DMSO, 187.6 µg, 0.38 µmol, 0.005 eq) were added to label the squarogels fluorescently. Subsequently, the pH-responsive ketal-crosslinker 2,2-bis(aminoethoxy)propane (1.8 µL, 11.3 µmol, 0.15 eq) was added and the resulting reaction mixture was stirred for 2 days at RT. To fabricate non-drug loaded fully PEGylated nanogels the remaining squaric ester amide units were reacted with $mPEG_{0.75kDa}$-NH_2. Therefore, an excess of

$mPEG_{0.75kDa}$-NH_2 stock solution (2 mL, 85 mg mL^{-1} in DMSO, 226.69 µmol, 170.01 mg, 3 eq.) was added and the reaction mixture was allowed to stir at RT for another 7 days. Complete conversion of the reactive ester units was confirmed by UV-Vis absorbance (compare Figure S6).

In order to purify the obtained squarogels and remove small molecular by-products, the reaction mixture was dialyzed (molecular weight cut-off: 1 kDa) against milliQ-water containing 0.1 % ammonia (1 L) for 7 days with frequent water exchanges. Subsequent lyophilization afforded the Oregon Green-labeled squarogels as fluffy orange powder (56 mg).

To ensure complete removal of residual unbound dye that could interfere with FCS measurements, the fluorescently labeled squarogels were further purified by spin-filtration. For this purpose, Oregon Green-labeled squarogels were redispersed in PBS (2 mg mL^{-1}) and purified using centrifugal filter units (regenerated cellulose, molecular weight cut-off: 10,000 g mol^{-1}). After each sequential centrifugation step PBS was added until complete disappearance of Oregon Green derived absorbance was observed by UV Vis measurements.

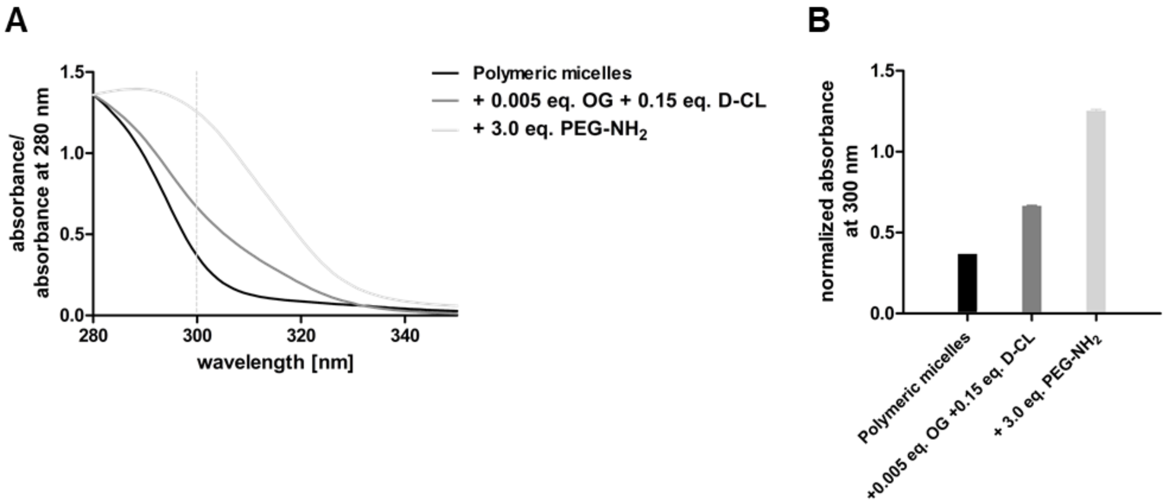

Figure S6. Conversion of polymeric squaric ester amides to squaric bisamides during preparation of OG-labeled pH-responsive nanogels monitored by UV absorbance. (A) UV-Vis spectra of precursor polymer after step-wise squarogel preparation in ethanol and (B) their corresponding absorbance at 300 nm.

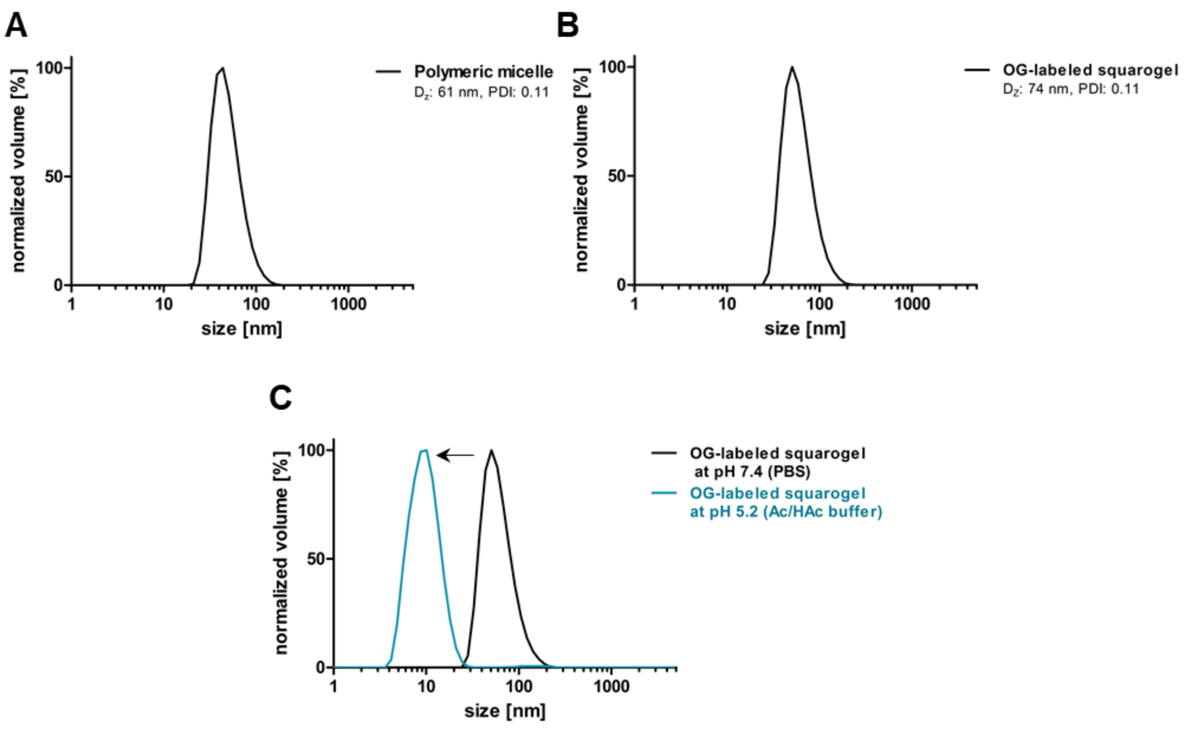

Figure S7. DLS size distribution of polymeric precursor micelles in ethanol (A), fully fabricated OG-labeled squarogels after re-dispersion in PBS (B) as well as after exposure to mildly acidic pH in Ac/HAc buffer compared to neutral pH in PBS (C).

3. FCS incubation experiments

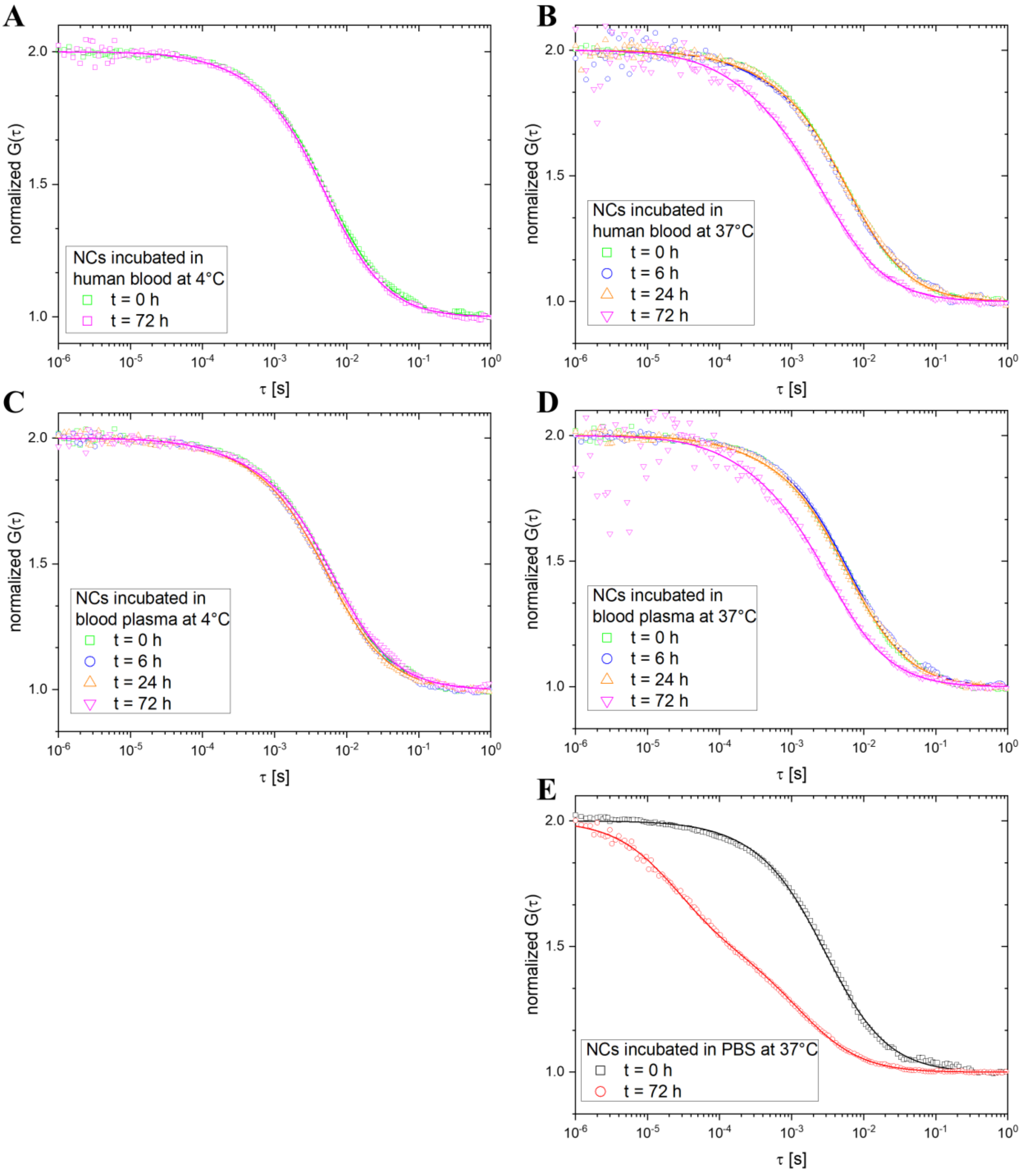

Figure S8. Normalized FCS autocorrelation curves of NCs incubated at 4°C in (**A**) human blood and (**C**) human blood plasma. Normalized FCS autocorrelation curves of NCs incubated at 37°C in (**B**) human blood, (**D**) human blood plasma and (**E**) PBS.

4. References

1. Huppertsberg, A.; Kaps, L.; Zhong, Z.; Schmitt, S.; Stickdorn, J.; Deswarte, K.; Combes, F.; Czysch, C.; De Vrieze, J.; Kasmi, S.; Choteschovsky, N.; Klefenz, A.; Medina-Montano, C.; Winterwerber, P.; Chen, C.; Bros, M.; Lienenklaus, S.; Sanders, N. N.; Koynov, K.; Schuppan, D.; Lambrecht, B. N.; David, S. A.; De Geest, B. G.; Nuhn, L., Squaric Ester-Based, pH-Degradable Nanogels: Modular Nanocarriers for Safe, Systemic Administration of Toll-like Receptor 7/8 Agonistic Immune Modulators. *J. Am. Chem. Soc.* **2021**, *143* (26), 9872–9883.
